# Bis[(4-cyano­benz­yl)ammonium] bis­(perchlorate) monohydrate

**DOI:** 10.1107/S1600536811048884

**Published:** 2011-11-25

**Authors:** Yao Huang

**Affiliations:** a School of Chemical Engineering, Yunnan Radio and TV University, Kunming 650023, People’s Republic of China

## Abstract

The asymmetric unit of the title compound, 2C_8_H_9_N_2_^+^·2ClO_4_^−^·H_2_O, consists of two (4-cyano­benz­yl)>ammonium cations, two disordered ClO_4_^−^ anions and one water mol­ecule. The differences in the two cations are reflected in the N—C—C—C torsion angles [−94.7 (3) and −115.9 (3)°]. In addition, the cations show different hydrogen-bonding patterns as one N atom bonds to two O atoms of ClO_4_^−^ ions, while the other N atom is involved in hydrogen bonding with the O atoms of the ClO_4_^−^ ions and water mol­ecules. In the crystal, N—H⋯O and O—H⋯O hydrogen bonds result in a three-dimensional network. An O atom in each of the anions is disordered over two positions of equal occupancy.

## Related literature

For a related structure, see: Shahwar *et al.* (2009 ▶).
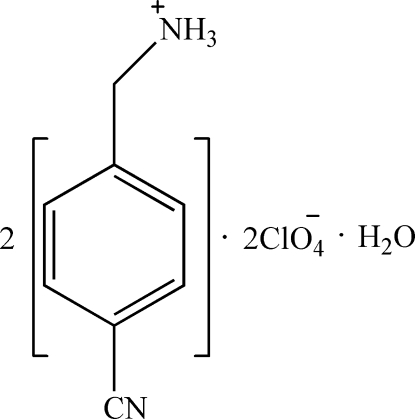


## Experimental

### 

#### Crystal data

2C_8_H_9_N_2_^+^·2ClO_4_^−^·H_2_O
*M* *_r_* = 483.26
Triclinic, 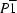

*a* = 5.0305 (10) Å
*b* = 13.616 (3) Å
*c* = 16.695 (3) Å
α = 66.79 (3)°
β = 86.17 (3)°
γ = 88.97 (3)°
*V* = 1048.6 (4) Å^3^
*Z* = 2
Mo *K*α radiation
μ = 0.37 mm^−1^
*T* = 293 K
0.29 × 0.25 × 0.21 mm

#### Data collection

Rigaku SCXmini diffractometer
Absorption correction: multi-scan (*CrystalClear*; Rigaku, 2005 ▶) *T*_min_ = 0.8, *T*_max_ = 0.9
10950 measured reflections
4796 independent reflections
3490 reflections with *I* > 2σ(*I*)
*R*_int_ = 0.038

#### Refinement

*R*[*F* ^2^ > 2σ(*F* ^2^)] = 0.066
*wR*(*F* ^2^) = 0.179
*S* = 1.00
4796 reflections
285 parameters
62 restraints
H atoms treated by a mixture of independent and constrained refinement
Δρ_max_ = 0.70 e Å^−3^
Δρ_min_ = −0.70 e Å^−3^

### 

Data collection: *CrystalClear* (Rigaku, 2005 ▶); cell refinement: *CrystalClear*; data reduction: *CrystalClear*; program(s) used to solve structure: *SHELXS97* (Sheldrick, 2008 ▶); program(s) used to refine structure: *SHELXL97* (Sheldrick, 2008 ▶); molecular graphics: *SHELXTL* (Sheldrick, 2008 ▶); software used to prepare material for publication: *SHELXL97*.

## Supplementary Material

Crystal structure: contains datablock(s) I, global. DOI: 10.1107/S1600536811048884/pv2463sup1.cif

Structure factors: contains datablock(s) I. DOI: 10.1107/S1600536811048884/pv2463Isup2.hkl

Supplementary material file. DOI: 10.1107/S1600536811048884/pv2463Isup3.cml

Additional supplementary materials:  crystallographic information; 3D view; checkCIF report

## Figures and Tables

**Table 1:** Hydrogen-bond geometry (Å, °)

*D*—H⋯*A*	*D*—H	H⋯*A*	*D*⋯*A*	*D*—H⋯*A*
N2—H2*C*⋯O1^i^	0.89	2.32	2.77 (3)	111
N2—H2*C*⋯O2^ii^	0.89	2.34	3.015 (4)	133
N2—H2*C*⋯O1′^i^	0.89	2.42	2.96 (3)	120
N2—H2*C*⋯O5′^i^	0.89	2.46	2.905 (9)	112
N2—H2*C*⋯O5^i^	0.89	2.55	3.036 (9)	115
N2—H2*E*⋯O7^iii^	0.89	2.43	3.306 (5)	167
N2—H2*E*⋯O5^iii^	0.89	2.45	3.176 (8)	139
N4—H4*C*⋯O1*W*^iv^	0.89	1.96	2.845 (4)	177
N4—H4*D*⋯O3^iv^	0.89	2.15	3.002 (5)	159
O1*W*—H1*WA*⋯O6^v^	0.82 (1)	2.02 (1)	2.839 (5)	173 (5)
O1*W*—H1*WB*⋯O7^ii^	0.82 (1)	2.21 (2)	2.991 (4)	160 (4)
O1*W*—H1*WB*⋯Cl2^ii^	0.82 (1)	2.98 (3)	3.593 (3)	133 (4)
N2—H2*D*⋯O2	0.89	2.25	3.096 (5)	158
N2—H2*D*⋯O3	0.89	2.40	3.154 (5)	143
N4—H4*B*⋯O1*W*	0.89	2.25	3.011 (4)	143
N4—H4*B*⋯O1′	0.89	2.50	3.04 (3)	119
N4—H4*B*⋯O5′	0.89	2.66	3.225 (8)	123
N4—H4*D*⋯O4	0.89	2.63	3.202 (5)	123

Symmetry codes: (i) 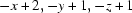; (ii) 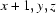; (iii) 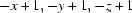; (iv) 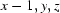; (v) 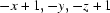.

**Fig. 1.**
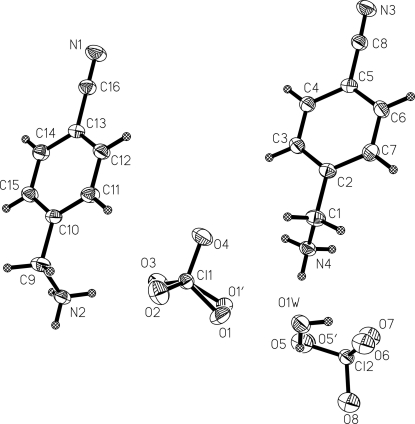
The asymmetric unit of the title compound with atom labels. Displacement ellipsoids were drawn at the 30% probability level. The oxygen atoms of the anions O1/O1' and O5/O5' were disordered in a 1:1 ratio.

**Fig. 2.**
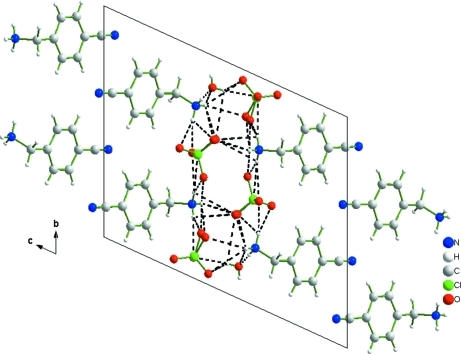
The packing viewed along the a-axis. Hydrogen bonds are drawn as dashed lines

## supplementary crystallographic information

### Comment

Recently, the crystal structure phenylmethanaminium chloroacetate (Shahwar *et al.*, 2009) has been reported. In our laboratory, a compound containing protonated 4-(aminomethyl)benzonitrile, ClO_4_^-^ anions and water molecule has been synthesized. In this paper, we report the crystal structure of the title compound.

The asymmetric unit of the title compound, consists of two independent 4-(aminomethyl)benzonitrile cations, two disorder ClO_4_^-^ anions and a water molecule. The dihedral angle between the 4-(aminomethyl)benzonitrile planes in the two cations is 7.25(9 )°. The atoms N2 and N4 are displaced out of these planes (C9–C16/N1 and C1–C8/N3) by 1.93 (3) and 1.325 (3) Å, respectively, with torsion angles C9/C12—C14/N2 and C6/C5—C2/N4 being -94.7 (3) and -115.9 (3) °, respectively.

The cations show different hydrogen bonding patterns as N2 atom bonds to two O atoms of ClO_4_^-^ ions, while N2 atom is involved in hydrogen bonding with the O atoms of the ClO_4_^-^ ions and H_2_O of hydration. In the crystal structure, intermolecular hydrogen bonds of the types N—H···O and O—H···O result in a three-dimensional network thus stabilizing the structure. (Tab. 1 & Fig. 2).

### Experimental

4-(Aminomethyl)benzonitrile (20 mmol, 2.64 g) and 10% aqueous HClO_4_ in a molar ratio of 1:1 were mixed and dissolved in 50 ml water by heating to 363 K forming a clear solution. The reaction mixture was cooled slowly to room temperature, block crystals of the title compound were formed after five days.

### Refinement

The H atoms were placed in calculated positions, with C—H = 0.93–0.97 Å and N—H = 0.89 Å, and refined using a riding model, with *U*_iso_(H) = 1.2*U*_eq_(C) or 1.5*U*_eq_(N). The H-atoms of water molecule were located from a difference Fourier map and were allowed to refine with O—H = 0.82 (1) Å constrains. For the disordered ClO_4_^-^, SIMU and DELU commands in SHELXL-97 (Sheldrick, 2008) were used to restrict their *U*_eq_.

### Crystal data

**Table:**

2C_8_H_9_N_2_^+^·2ClO_4_^−^·H_2_O	*Z* = 2
*M**_r_* = 483.26	*F*(000) = 500
Triclinic, *P*1	*D*_x_ = 1.531 Mg m^−^^3^
Hall symbol: -P 1	Mo *K*α radiation, λ = 0.71073 Å
*a* = 5.0305 (10) Å	Cell parameters from 3490 reflections
*b* = 13.616 (3) Å	θ = 3.3–27.5°
*c* = 16.695 (3) Å	µ = 0.37 mm^−^^1^
α = 66.79 (3)°	*T* = 293 K
β = 86.17 (3)°	Block, colorless
γ = 88.97 (3)°	0.29 × 0.25 × 0.21 mm
*V* = 1048.6 (4) Å^3^	

### Data collection

**Table:**

Rigaku SCXmini diffractometer	4796 independent reflections
Radiation source: fine-focus sealed tube	3490 reflections with *I* > 2σ(*I*)
graphite	*R*_int_ = 0.038
Detector resolution: 13.6612 pixels mm^-1^	θ_max_ = 27.5°, θ_min_ = 3.3°
ω scans	*h* = −6→6
Absorption correction: multi-scan (*CrystalClear*; Rigaku, 2005)	*k* = −17→17
*T*_min_ = 0.8, *T*_max_ = 0.9	*l* = −21→21
10950 measured reflections	

### Refinement

**Table:**

Refinement on *F*^2^	Secondary atom site location: difference Fourier map
Least-squares matrix: full	Hydrogen site location: inferred from neighbouring sites
*R*[*F*^2^ > 2σ(*F*^2^)] = 0.066	H atoms treated by a mixture of independent and constrained refinement
*wR*(*F*^2^) = 0.179	*w* = 1/[σ^2^(*F*_o_^2^) + (0.0699*P*)^2^ + 1.794*P*] where *P* = (*F*_o_^2^ + 2*F*_c_^2^)/3
*S* = 1.00	(Δ/σ)_max_ = 0.001
4796 reflections	Δρ_max_ = 0.70 e Å^−^^3^
285 parameters	Δρ_min_ = −0.70 e Å^−^^3^
62 restraints	Extinction correction: *SHELXL97* (Sheldrick, 2008), Fc^*^=kFc[1+0.001xFc^2^λ^3^/sin(2θ)]^-1/4^
Primary atom site location: structure-invariant direct methods	Extinction coefficient: 0.047 (4)

### Special details

**Table:**

Geometry. All e.s.d.'s (except the e.s.d. in the dihedral angle between two l.s. planes) are estimated using the full covariance matrix. The cell e.s.d.'s are taken into account individually in the estimation of e.s.d.'s in distances, angles and torsion angles; correlations between e.s.d.'s in cell parameters are only used when they are defined by crystal symmetry. An approximate (isotropic) treatment of cell e.s.d.'s is used for estimating e.s.d.'s involving l.s. planes.
Refinement. Refinement of *F*^2^ against ALL reflections. The weighted *R*-factor *wR* and goodness of fit *S* are based on *F*^2^, conventional *R*-factors *R* are based on *F*, with *F* set to zero for negative *F*^2^. The threshold expression of *F*^2^ > σ(*F*^2^) is used only for calculating *R*-factors(gt) *etc*. and is not relevant to the choice of reflections for refinement. *R*-factors based on *F*^2^ are statistically about twice as large as those based on *F*, and *R*- factors based on ALL data will be even larger.

### Fractional atomic coordinates and isotropic or equivalent isotropic displacement parameters (Å^2^)

**Table:**

	*x*	*y*	*z*	*U*_iso_*/*U*_eq_	Occ. (<1)
N1	0.5034 (8)	0.8947 (3)	−0.0455 (3)	0.0622 (10)	
N2	1.2660 (6)	0.6834 (3)	0.36264 (19)	0.0446 (7)	
H2C	1.3892	0.6573	0.4013	0.067*	
H2D	1.1463	0.6327	0.3702	0.067*	
H2E	1.1855	0.7382	0.3702	0.067*	
C11	1.0956 (8)	0.6906 (3)	0.1705 (2)	0.0445 (9)	
H11A	1.1534	0.6202	0.1906	0.053*	
C15	1.1073 (8)	0.8638 (3)	0.1743 (2)	0.0431 (8)	
H15A	1.1737	0.9104	0.1966	0.052*	
C13	0.8291 (7)	0.8294 (3)	0.0775 (2)	0.0367 (7)	
C10	1.1925 (7)	0.7590 (3)	0.2048 (2)	0.0363 (7)	
C14	0.9249 (8)	0.8999 (3)	0.1109 (2)	0.0441 (8)	
H14A	0.8668	0.9702	0.0909	0.053*	
C9	1.3941 (7)	0.7202 (3)	0.2730 (2)	0.0474 (9)	
H9A	1.4938	0.6618	0.2666	0.057*	
H9B	1.5188	0.7777	0.2641	0.057*	
C16	0.6445 (8)	0.8667 (3)	0.0090 (2)	0.0444 (8)	
C12	0.9149 (8)	0.7251 (3)	0.1071 (2)	0.0443 (8)	
H12A	0.8510	0.6785	0.0844	0.053*	
N3	−0.4792 (8)	0.3873 (3)	−0.0191 (2)	0.0595 (9)	
N4	0.3279 (6)	0.2507 (3)	0.37842 (19)	0.0423 (7)	
H4B	0.4520	0.2284	0.4171	0.063*	
H4C	0.1783	0.2134	0.4011	0.063*	
H4D	0.2968	0.3197	0.3653	0.063*	
C3	0.1437 (8)	0.3734 (3)	0.1900 (2)	0.0444 (8)	
H3A	0.2187	0.4246	0.2058	0.053*	
C1	0.4220 (7)	0.2352 (3)	0.2980 (2)	0.0459 (9)	
H1A	0.4646	0.1605	0.3133	0.055*	
H1B	0.5839	0.2767	0.2733	0.055*	
C8	−0.3365 (8)	0.3600 (3)	0.0352 (2)	0.0433 (8)	
C6	−0.0743 (8)	0.2218 (3)	0.1421 (3)	0.0458 (9)	
H6A	−0.1469	0.1706	0.1258	0.055*	
C2	0.2180 (7)	0.2681 (3)	0.2300 (2)	0.0371 (7)	
C7	0.1075 (8)	0.1923 (3)	0.2058 (3)	0.0464 (9)	
H7A	0.1560	0.1210	0.2326	0.056*	
C4	−0.0396 (8)	0.4042 (3)	0.1271 (2)	0.0438 (8)	
H4A	−0.0901	0.4753	0.1013	0.053*	
C5	−0.1485 (7)	0.3278 (3)	0.1025 (2)	0.0375 (7)	
Cl1	0.81260 (16)	0.45679 (7)	0.38569 (6)	0.0390 (2)	
O1	0.771 (5)	0.356 (3)	0.4610 (17)	0.068 (5)	0.50
O1'	0.709 (6)	0.363 (3)	0.4491 (18)	0.073 (5)	0.50
O2	0.7525 (6)	0.5448 (3)	0.4098 (2)	0.0673 (9)	
O3	1.0947 (6)	0.4613 (3)	0.3634 (2)	0.0665 (9)	
O4	0.6749 (6)	0.4664 (3)	0.3112 (2)	0.0647 (8)	
Cl2	0.23525 (17)	0.09462 (7)	0.62797 (6)	0.0409 (2)	
O6	0.3449 (6)	0.0479 (3)	0.5708 (2)	0.0705 (5)	
O7	−0.0484 (6)	0.0887 (3)	0.6317 (2)	0.0705 (5)	
O8	0.3295 (6)	0.0431 (3)	0.7120 (2)	0.0705 (5)	
O5	0.2648 (14)	0.2074 (7)	0.6001 (5)	0.0705 (5)	0.50
O5'	0.3568 (14)	0.1992 (7)	0.5835 (5)	0.0705 (5)	0.50
O1W	0.8375 (6)	0.1387 (3)	0.4461 (2)	0.0520 (7)	
H1WA	0.799 (10)	0.083 (2)	0.442 (3)	0.075 (17)*	
H1WB	0.827 (9)	0.128 (4)	0.4981 (8)	0.059 (14)*	

### Atomic displacement parameters (Å^2^)

**Table:**

	*U*^11^	*U*^22^	*U*^33^	*U*^12^	*U*^13^	*U*^23^
N1	0.070 (2)	0.057 (2)	0.063 (2)	0.0166 (18)	−0.035 (2)	−0.0242 (18)
N2	0.0431 (17)	0.0548 (19)	0.0367 (16)	−0.0024 (14)	−0.0109 (13)	−0.0175 (14)
C11	0.053 (2)	0.0374 (19)	0.044 (2)	0.0122 (16)	−0.0147 (17)	−0.0160 (16)
C15	0.050 (2)	0.0426 (19)	0.0417 (19)	0.0012 (16)	−0.0097 (16)	−0.0209 (16)
C13	0.0365 (17)	0.0427 (19)	0.0316 (17)	0.0051 (14)	−0.0069 (13)	−0.0150 (14)
C10	0.0326 (17)	0.0454 (19)	0.0301 (16)	0.0022 (14)	−0.0032 (13)	−0.0138 (14)
C14	0.052 (2)	0.0365 (19)	0.045 (2)	0.0070 (16)	−0.0091 (16)	−0.0166 (16)
C9	0.0368 (19)	0.067 (3)	0.0360 (19)	0.0068 (17)	−0.0091 (15)	−0.0168 (18)
C16	0.047 (2)	0.043 (2)	0.044 (2)	0.0073 (16)	−0.0117 (17)	−0.0164 (16)
C12	0.054 (2)	0.0408 (19)	0.043 (2)	0.0017 (16)	−0.0142 (17)	−0.0193 (16)
N3	0.069 (2)	0.060 (2)	0.057 (2)	0.0119 (18)	−0.0292 (19)	−0.0274 (18)
N4	0.0353 (15)	0.0513 (18)	0.0396 (16)	−0.0022 (13)	−0.0093 (12)	−0.0159 (14)
C3	0.050 (2)	0.0411 (19)	0.046 (2)	−0.0003 (16)	−0.0115 (16)	−0.0202 (16)
C1	0.0342 (18)	0.060 (2)	0.044 (2)	0.0085 (16)	−0.0076 (15)	−0.0213 (18)
C8	0.047 (2)	0.045 (2)	0.042 (2)	0.0020 (16)	−0.0067 (17)	−0.0199 (17)
C6	0.050 (2)	0.043 (2)	0.051 (2)	−0.0010 (16)	−0.0124 (17)	−0.0240 (17)
C2	0.0331 (17)	0.047 (2)	0.0338 (17)	0.0030 (14)	−0.0021 (13)	−0.0184 (15)
C7	0.053 (2)	0.0351 (19)	0.051 (2)	0.0074 (16)	−0.0103 (17)	−0.0154 (16)
C4	0.051 (2)	0.0361 (18)	0.044 (2)	0.0040 (16)	−0.0105 (16)	−0.0151 (16)
C5	0.0357 (18)	0.0453 (19)	0.0314 (16)	0.0024 (14)	−0.0042 (13)	−0.0149 (15)
Cl1	0.0360 (4)	0.0405 (5)	0.0415 (5)	0.0010 (3)	−0.0033 (3)	−0.0172 (4)
O1	0.090 (12)	0.056 (5)	0.042 (8)	−0.005 (7)	−0.005 (8)	−0.004 (6)
O1'	0.100 (12)	0.061 (8)	0.043 (6)	−0.033 (8)	−0.028 (6)	0.000 (5)
O2	0.0622 (19)	0.066 (2)	0.094 (2)	0.0170 (15)	−0.0153 (17)	−0.0525 (19)
O3	0.0370 (15)	0.077 (2)	0.095 (2)	0.0056 (14)	−0.0030 (15)	−0.0434 (19)
O4	0.0577 (18)	0.087 (2)	0.0553 (18)	−0.0067 (16)	−0.0151 (14)	−0.0324 (17)
Cl2	0.0403 (5)	0.0452 (5)	0.0388 (5)	−0.0029 (4)	−0.0029 (3)	−0.0181 (4)
O6	0.0604 (11)	0.0881 (12)	0.0651 (11)	0.0018 (9)	−0.0026 (8)	−0.0325 (9)
O7	0.0604 (11)	0.0881 (12)	0.0651 (11)	0.0018 (9)	−0.0026 (8)	−0.0325 (9)
O8	0.0604 (11)	0.0881 (12)	0.0651 (11)	0.0018 (9)	−0.0026 (8)	−0.0325 (9)
O5	0.0604 (11)	0.0881 (12)	0.0651 (11)	0.0018 (9)	−0.0026 (8)	−0.0325 (9)
O5'	0.0604 (11)	0.0881 (12)	0.0651 (11)	0.0018 (9)	−0.0026 (8)	−0.0325 (9)
O1W	0.0477 (16)	0.0612 (19)	0.0485 (17)	−0.0074 (14)	−0.0066 (13)	−0.0222 (15)

### Geometric parameters (Å, °)

**Table:**

N1—C16	1.132 (5)	C3—H3A	0.9300
N2—C9	1.482 (5)	C1—C2	1.510 (5)
N2—H2C	0.8900	C1—H1A	0.9700
N2—H2D	0.8900	C1—H1B	0.9700
N2—H2E	0.8900	C8—C5	1.444 (5)
C11—C12	1.374 (5)	C6—C7	1.382 (5)
C11—C10	1.380 (5)	C6—C5	1.386 (5)
C11—H11A	0.9300	C6—H6A	0.9300
C15—C14	1.381 (5)	C2—C7	1.384 (5)
C15—C10	1.384 (5)	C7—H7A	0.9300
C15—H15A	0.9300	C4—C5	1.392 (5)
C13—C12	1.379 (5)	C4—H4A	0.9300
C13—C14	1.393 (5)	Cl1—O1'	1.38 (3)
C13—C16	1.447 (5)	Cl1—O4	1.423 (3)
C10—C9	1.506 (5)	Cl1—O2	1.428 (3)
C14—H14A	0.9300	Cl1—O3	1.439 (3)
C9—H9A	0.9700	Cl1—O1	1.45 (3)
C9—H9B	0.9700	Cl2—O8	1.410 (3)
C12—H12A	0.9300	Cl2—O6	1.420 (3)
N3—C8	1.135 (5)	Cl2—O5	1.426 (9)
N4—C1	1.486 (5)	Cl2—O7	1.426 (3)
N4—H4B	0.8900	Cl2—O5'	1.445 (9)
N4—H4C	0.8900	O5—O5'	0.557 (8)
N4—H4D	0.8900	O1W—H1WA	0.820 (2)
C3—C4	1.377 (5)	O1W—H1WB	0.820 (2)
C3—C2	1.379 (5)		
			
C9—N2—H2C	109.5	N4—C1—H1B	109.1
C9—N2—H2D	109.5	C2—C1—H1B	109.1
H2C—N2—H2D	109.5	H1A—C1—H1B	107.8
C9—N2—H2E	109.5	N3—C8—C5	178.1 (5)
H2C—N2—H2E	109.5	C7—C6—C5	119.9 (3)
H2D—N2—H2E	109.5	C7—C6—H6A	120.0
C12—C11—C10	120.8 (3)	C5—C6—H6A	120.0
C12—C11—H11A	119.6	C3—C2—C7	119.3 (3)
C10—C11—H11A	119.6	C3—C2—C1	120.8 (3)
C14—C15—C10	120.6 (3)	C7—C2—C1	119.9 (3)
C14—C15—H15A	119.7	C6—C7—C2	120.4 (3)
C10—C15—H15A	119.7	C6—C7—H7A	119.8
C12—C13—C14	120.5 (3)	C2—C7—H7A	119.8
C12—C13—C16	119.6 (3)	C3—C4—C5	119.4 (3)
C14—C13—C16	119.8 (3)	C3—C4—H4A	120.3
C11—C10—C15	119.4 (3)	C5—C4—H4A	120.3
C11—C10—C9	120.1 (3)	C6—C5—C4	119.8 (3)
C15—C10—C9	120.5 (3)	C6—C5—C8	120.5 (3)
C15—C14—C13	119.0 (3)	C4—C5—C8	119.6 (3)
C15—C14—H14A	120.5	O1'—Cl1—O4	102.7 (9)
C13—C14—H14A	120.5	O1'—Cl1—O2	110.4 (14)
N2—C9—C10	111.8 (3)	O4—Cl1—O2	110.4 (2)
N2—C9—H9A	109.3	O1'—Cl1—O3	117.5 (13)
C10—C9—H9A	109.3	O4—Cl1—O3	108.8 (2)
N2—C9—H9B	109.3	O2—Cl1—O3	106.82 (19)
C10—C9—H9B	109.3	O1'—Cl1—O1	14.8 (18)
H9A—C9—H9B	107.9	O4—Cl1—O1	115.4 (10)
N1—C16—C13	178.5 (4)	O2—Cl1—O1	110.3 (11)
C11—C12—C13	119.6 (3)	O3—Cl1—O1	104.6 (11)
C11—C12—H12A	120.2	O8—Cl2—O6	110.8 (2)
C13—C12—H12A	120.2	O8—Cl2—O5	109.5 (4)
C1—N4—H4B	109.5	O6—Cl2—O5	117.2 (3)
C1—N4—H4C	109.5	O8—Cl2—O7	110.7 (2)
H4B—N4—H4C	109.5	O6—Cl2—O7	109.4 (2)
C1—N4—H4D	109.5	O5—Cl2—O7	98.7 (3)
H4B—N4—H4D	109.5	O8—Cl2—O5'	110.7 (4)
H4C—N4—H4D	109.5	O6—Cl2—O5'	97.5 (3)
C4—C3—C2	121.1 (3)	O5—Cl2—O5'	22.3 (3)
C4—C3—H3A	119.4	O7—Cl2—O5'	117.1 (3)
C2—C3—H3A	119.4	O5'—O5—Cl2	80.8 (15)
N4—C1—C2	112.7 (3)	O5—O5'—Cl2	76.9 (15)
N4—C1—H1A	109.1	H1WA—O1W—H1WB	108 (5)
C2—C1—H1A	109.1		

### Hydrogen-bond geometry (Å, °)

**Table:**

*D*—H···*A*	*D*—H	H···*A*	*D*···*A*	*D*—H···*A*
N2—H2C···O1^i^	0.89	2.32	2.77 (3)	111.
N2—H2C···O2^ii^	0.89	2.34	3.015 (4)	133.
N2—H2C···O1'^i^	0.89	2.42	2.96 (3)	120.
N2—H2C···O5'^i^	0.89	2.46	2.905 (9)	112.
N2—H2C···O5^i^	0.89	2.55	3.036 (9)	115.
N2—H2E···O7^iii^	0.89	2.43	3.306 (5)	167.
N2—H2E···O5^iii^	0.89	2.45	3.176 (8)	139.
N4—H4C···O1W^iv^	0.89	1.96	2.845 (4)	177.
N4—H4D···O3^iv^	0.89	2.15	3.002 (5)	159.
O1W—H1WA···O6^v^	0.82 (1)	2.02 (1)	2.839 (5)	173 (5)
O1W—H1WB···O7^ii^	0.82 (1)	2.21 (2)	2.991 (4)	160 (4)
O1W—H1WB···Cl2^ii^	0.82 (1)	2.98 (3)	3.593 (3)	133 (4)
N2—H2D···O2	0.89	2.25	3.096 (5)	158.
N2—H2D···O3	0.89	2.40	3.154 (5)	143.
N4—H4B···O1W	0.89	2.25	3.011 (4)	143.
N4—H4B···O1'	0.89	2.50	3.04 (3)	119.
N4—H4B···O5'	0.89	2.66	3.225 (8)	123.
N4—H4D···O4	0.89	2.63	3.202 (5)	123.

Symmetry codes: (i) −*x*+2, −*y*+1, −*z*+1; (ii) *x*+1, *y*, *z*; (iii) −*x*+1, −*y*+1, −*z*+1; (iv) *x*−1, *y*, *z*; (v) −*x*+1, −*y*, −*z*+1.
